# Safety and efficacy of intracoronary artery administration of human bone marrow-derived mesenchymal stem cells in STEMI of Lee-Sung pigs—A preclinical study for supporting the feasibility of the OmniMSC-AMI phase I clinical trial

**DOI:** 10.3389/fcvm.2023.1153428

**Published:** 2023-03-29

**Authors:** Wannhsin Chen, Chun-Hsiang Hou, Yi-Ling Chen, Hsin-Hsin Shen, Chen-Hsuan Lin, Cheng-Yi Wu, Meng-Hsueh Lin, Chih-Ching Liao, Jun-Jae Huang, Chi-Yu Yang, Yi-Chen Li, Hon-Kan Yip

**Affiliations:** ^1^ Regeneration Medicine Technology Division, Biomedical Technology and Device Research Laboratories, Industrial Technology Research Institute, Hsinchu, Taiwan; ^2^ Animal Technology Laboratories, Agricultural Technology Research Institute, Miaoli, Taiwan; ^3^ Division of Cardiology, Department of Internal Medicine, Kaohsiung Chang Gung Memorial Hospital and Chang Gung University College of Medicine, Kaohsiung, Taiwan; ^4^ Institute for Translational Research in Biomedicine, Kaohsiung Chang Gung Memorial Hospital, Kaohsiung, Taiwan; ^5^ Center of Cell Therapy, National Cheng Kung University Hospital, College of Medicine, National Cheng Kung University, Tainan, Taiwan; ^6^ Clinical Medicine Research Center, National Cheng Kung University Hospital, College of Medicine, National Cheng Kung University, Tainan, Taiwan; ^7^ Institute of Clinical Medicine, College of Medicine, National Cheng Kung University, Tainan, Taiwan; ^8^ Center for Shockwave Medicine and Tissue Engineering, Kaohsiung Chang Gung Memorial Hospital, Kaohsiung, Taiwan; ^9^ School of Medicine, College of Medicine, Chang Gung University, Taoyuan, Taiwan; ^10^ Department of Medical Research, China Medical University Hospital, China Medical University, Taichung, Taiwan; ^11^ Department of Nursing, Asia University, Taichung, Taiwan; ^12^ Division of Cardiology, Department of Internal Medicine, Xiamen Chang Gung Hospital, Xiamen, China

**Keywords:** acute myocardial infarction, balloon occlusion, exogenic mesenchymal stem cells, porcine, left ventricular ejection fraction, fibrosis

## Abstract

**Background:**

This study tested whether early left intracoronary arterial (LAD) administration of human bone marrow-derived mesenchymal stem cells (hBMMSCs, called OmniMSCs) in acute ST-segment elevation myocardial infarction (STEMI) of Lee-Sung pigs induced by 90 min balloon-occluded LAD was safe and effective.

**Methods and results:**

Young male Lee-Sung pigs were categorized into SC (sham-operated control, *n* = 3), AMI-B (STEMI + buffer/21 cc/administered at 90 min after STEMI, *n* = 6), and AMI-M [acute myocardial infarction (AMI) + hBMMSCs/1.5 × 10^7^/administered at 90 min after STEMI, *n* = 6] groups. By 2 and 5 months after STEMI, the cardiac magnetic resonance imaging demonstrated that the muscle scar score (MSS) and abnormal cardiac muscle exercise score in the infarct region were significantly increased in the AMI-B than in the SC group that were significantly reversed in the AMI-M group, whereas the left ventricular ejection function by each month (from 1 to 5) displayed an opposite pattern of MSS among the groups (all *p* < 0.001). By 5 months, histopathological findings of infarct and fibrosis areas and isolectin-B4 exhibited an identical pattern, whereas the cellular expressions of troponin-I/troponin-T/von Willebrand factor exhibited an opposite pattern of MSS among the groups (all *p* < 0.001). The ST-segment resolution (>80%) was significantly earlier (estimated after 6-h AMI) in the AMI-M group than in the AMI-B group (*p* < 0.001). The protein expressions of inflammation (IL-1β/TNF-α/NF-κB)/oxidative stress (NOX-1/NOX-2/oxidized protein)/apoptosis (cleaved caspase-3/cleaved PARP)/DNA damage (γ-H2AX) displayed an identical pattern to MSS among the groups, whereas the protein expressions of angiogenesis factors (SDF-1α/VEGF) were significantly and progressively increased from SC, AMI-B, to AMI-M groups (all *p* < 0.001).

**Conclusion:**

Early intra-LAD transfusion of OmniMSC treatment effectively reduced the infarct size and preserved LV function in porcine STEMI.

## Introduction

Early and prompt mechanical restoration of the normal blood flow in the infarct-related artery (IRA) is the fundamental and universal concept for treatment of acute ST-segment elevation myocardial infarction (STEMI) (1–4). Currently, it is universally accepted that primary percutaneous coronary intervention (primary PCI) is one of the best and most popular methods for treatment of STEMI (1–6) because this strategic management results in greater than 95% of the IRA to quickly achieve normal blood flow and, therefore, salvage the dying myocardium, and subsequently improve the left ventricular (LV) function (1, 2, 4, 7, 8).

However, pump failure (i.e., mechanical) and heart failure (i.e., clinical) caused by losing myocardium/contractility and, in particular, death are commonly reported worldwide in patients with acute myocardial infarction (AMI) even undergoing primary PCI (9–14), suggesting that the treatment of AMI has many unmet needs (1, 15, 16). Accordingly, to find a new comprehensive modality with safety and efficacy is of paramount importance for cardiologists and AMI patients. However, prior to finding such an innovative method for subsequent treatment of AMI just after the primary PCI procedure, the pathophysiological mechanism of myocardial damage/death prior to and after primary PCI should be more thoroughly clarified (4). According to the reports from basic and clinical research (1, 4, 17–24), it is reasonable to believe that at least six cardinal reasons for myocardial damage: (1) myocardial necrosis due to complete loss of the blood supply prior to primary PCI, (2) ischemia-reperfusion injury, (3) AMI-elicited rigorous inflammatory reaction, (4) immune hyper-reactivity (i.e., overwhelming immune response), (5) inflammatory cell infiltration and proinflammatory cytokine release in infarct/peri-infarct areas, and (6) generations of reactive oxygen species (ROS)/free radicals which cause oxidative stress after AMI.

Growing evidence has demonstrated that the mesenchymal stem cells (MSCs) have capacity of anti-inflammation, immunomodulation, downregulation of oxidative stress, upregulation of angiogenesis, and tissue regeneration (25–28). Additionally, animal model studies have recently demonstrated that MSC therapy effectively protected the heart function against AMI damage (29–31). Our previous studies have also demonstrated that early administration of MSCs (i.e., ≤3 h after AMI induction) for either small or large AMI animals significantly improved the left ventricular ejection fraction (LVEF) mainly through anti-inflammation, immunomodulation, and downregulation of oxidative stress (25–27). However, the limitation of these studies (25–27) was that the route of MSC administration was through the opening the chest wall and directly implanted MSCs into the LV myocardium, suggesting that it was an invasive procedure with a high degree of risk, which might be harmful to animals. On the other hand, when looking into previous clinical trials (32–39) of stem cell therapy for AMI patients, we found that although most of these trials were safe, their efficacy was universally inconsistent. These results raise the issue that there might be confounders such as (1) too late administration of MSCs, i.e., >3–5 days to 30 days after AMI, suggesting the myocardium already in complete necrosis/death that was against the concept of early and prompt treatment of STEMI; (2) intravenous administration of the MSCs rather than catheter-based cell therapy (i.e., *via* catheter-intracoronary transfusion); and (3) inadequate number of MSCs to be utilized for some patients because MSCs being autologous by which intrinsic self-expansion was usually impaired in patients with AMI and coronary artery disease (CAD).

Based on the above-mentioned issues (25–39), we cooperated with the Industrial Technology Research Institute (ITRI) in Taiwan, which established many Good tissue practice (GTP)-compliant MSC banks suitable for clinical application, to perform a preclinical study addressing the safety and efficacy of early intracoronary artery administration of human bone marrow-derived mesenchymal stem cells (hBMMSCs) in porcine AMI models. Based on the results of this preclinical study, the Taiwan Food and Drug Administration (TFDA) granted a phase I clinical trial entitled “Intracoronary administration of OmniMSC-AMI for acute STEMI patients undergoing primary PCI,” i.e., a phase I clinical trial to assess the safety of the OmniMSC-AMI therapy [IRB number: 202002401A0 and TFDA number: 10696614980]. In this clinical trial, the novel strategic management is that allogenic hBMMSCs, i.e., the product called OmniMSC-AMI, were administered *via* guiding a catheter just at the time of completion of primary PCI procedure. Herein, we reported the results of this preclinical study.

## Materials and methods

### Ethics

All animal procedures were approved by the Agricultural Technology Research Institute (Hsinchu City, Taiwan) (Affidavit of Approval of Animal Use Protocol No. 107121C2) and performed in accordance with the Guide for the Care and Use of Laboratory Animals. Animals were housed in an Association for Assessment and Accreditation of Laboratory Animal Care International (AAALAC; Frederick, MD, United States)-approved animal facility in Agricultural Technology Research Institute with controlled temperature and light cycles (22°C and 12/12 light cycle).

### Procedure and protocol of acute STEMI in Lee-Sung pigs and animal grouping

To mimic the clinical setting of STEMI in human beings, pathogen-free male Lee-Sung pigs (Agricultural Technology Research Institute, Hsinchu City, Taiwan) (*n* = 15) weighing 25–30 kg were used for the present study. Prior to induction of acute STEMI, the animals were fed for 1 week for adapting with the environment.

In detail, animals were anesthetized by inhalational 2.0% isoflurane and placed in the prone position on a warming pad at 37°C for laminectomy. Animals in the sham-operated control (SC) group underwent opening of skin and muscle layers only, while AMI groups received a complete AMI induction procedure. The procedures are described step-by-step in detail below:

1. Lee-Sung pigs were anesthetized by inhalation of 2.0% isoflurane. The animals were then placed on a prone position at a warming pad at 37°C to secure the head and shave the right neck hair.
2. Skin incision was carried out, followed by carefully dissecting and separating the muscle layers at the neck carotid triangle area.
3. The distal portion of the right common carotid artery (RCCA) was first permanently ligated by the suture line.
4. The RCCA was identified and isolated, followed by incision of a small hole with knife, and the wire was put into the hole and advanced into the proximal portion of the RCCA. Finally, a 6-French artery sheath was inserted into the proximal portion of RCCA along the wire. These above-mentioned procedures were designed for the purpose of creating an access route for the cardiac catheterization procedure.

For the sham-operated control group (i.e., SC group), the animals (*n* = 3) just received the buffer (21 mL) without balloon occlusion, followed by closure of skin and muscle layers of the right neck. For the STEMI induction (*n* = 12), animals were equally categorized into AMI-B [AMI + buffer (21 mL)] and AMI-M [AMI + hBMMSCs (1.5 × 10^7^ cells)], respectively.

### Procedure and protocol for AMI induction and hBMMSC treatment

Under anesthesia, the 6-French JL3.5 guiding catheter (Terumo) was inserted into the arterial sheath and advanced into the ascending aorta. The guiding catheter was then engaged into the left main trunk. Under the fluoroscope, the 0.014-inch PCI guidewire (Terumo) was advanced into the distal left coronary artery (LAD) through the guiding catheter. A suitable size PCI balloon was then advanced along with the wire into the LAD just beyond the level of the first diagonal branch. Under the fluoroscope, the balloon was inflated up to an appropriate size, i.e., to completely occlude the blood flow in the distal LAD that was identified by injection of the contrast media into the LAD. The total occlusion time was 90 min, followed by deflating the balloon and removing it from the guiding catheter for LAD reperfusion. During the procedure, electrocardiography (ECG) was monitored continuously and the ECG signaling change was completely recorded, i.e., for confirming the STEMI induction. The 12-lead ECG was serially performed for the animals, i.e., at the baseline and at 30- and 90 min during LAD occlusion.

By 90 min AMI induction, the frozen hBMMSCs (1.5 × 10^7 ^cells contained in normal saline 20 mL) (product name: OmniMSC) was thawed, and (>3.0 min) intracoronary transfusion was done slowly into the proximal portion of the LAD in the AMI-M group by a microcatheter. The dose of hBMMSCs to be utilized for one vessel in the present study was based on our previous report (40) with minimal modification. On the other hand, a frozen buffer solution (20 mL) was thawed and (>3.0 min) intracoronary transfusion was done slowly into the proximal portion of the LAD in the AMI-B group by a microcatheter. Finally, after buffer or stem cell therapy, the animals recovered from anesthesia and were intensively monitored for 24 h after the procedure. OmniMSC has been approved for utilization in phase I clinical trials by TFDA and IRB in our institute.

The 12-lead ECG was serially recorded at time points of 1.5, 3, 6, 24, and 48 h as well as at days 7, 30, 90, and 150 after 90 min AMI induction by ballooning, respectively.

### LV functional assessment by transthoracic echocardiography

The procedure and protocol have been depicted in our previous reports (25, 41). In detail, a transthoracic *two-dimensional* (2D) echocardiographic study was conducted in each group prior to and at the time points of 1.5 h, 3 h, and day 7 as well as in each month (i.e., 1–5 months) after STEMI induction. The procedure was conducted by an animal cardiologist blinded to the experimental design using an ultrasound machine (Siemens ACUSON Antares Ultrasound System). An M-*mode* standard 2D left parasternal-*long* axis echocardiographic examination was conducted. Left ventricular internal dimensions [end-systolic diameter (ESD) and end-diastolic diameter (EDD)] were measured at the mitral valve and papillary levels of the left ventricle, according to the American Society of Echocardiography leading-edge method using at least three consecutive cardiac cycles. LVEF was calculated as follows: LVEF (%) = [(LVEDD^3^ − LVESD^3^)/LVEDD^3^] × 100%.

### Cardiac MRI study

The protocol and procedure of magnetic resonance imaging (MRI) were based on our previous report (41). Briefly, a cardiac MRI study was performed for all animals at baseline and in 2 and 5 months after STEMI induction. The study was performed by one cardiac MRI expert blinded to the treatment protocol using a Philips Achieva 3.0T X-Series MRI machine. After anesthesia, with the Lee-Sung pigs in a supine position, all four limbs were fixed by Velcro strips. A phased array coil was wrapped around the chest. A cine MRI for assessment of the LV volume and functional integrity was conducted using a balanced steady-state free precession sequence with the following parameters: repetition time ms/echo time ms, 2.8/1.42 (2CH) and 3.1/1.56 (4CH); section thickness, 6 mm; spacing, 0; flip angle, 45°; field of view, 277 × 277 mm; matrix size, 122 × 116 (2CH) and 1,156 × 180 (4CH); number of signals acquired, one; and, number of phase per slice, one. The parameters of cardiac muscle exercise score and scar score were recorded in detail and calculated by software.

Animals were euthanized on day 150 (i.e., at the end of 5 months) after STEMI induction and the LV myocardium was harvested immediately for individual study.

### Definition of abnormality of cardiac muscle exercise score

Semiquantitative analysis was performed for the abnormal cardiac muscle exercise score in the infarct area, i.e., hypokinesis, akinesis, and dyskinesis, in the present study. Abnormal cardiac muscle exercise was categorized into scores as 3 = dyskinesia, 2 = akinesia, and 1 = hypokinesia. In detail, akinesia was defined as lack of movement or contraction in the infarcted region of the heart muscle; dyskinesia corresponded to an abnormal movement instead of contracting in systole and the segment of myocardium bulges out during LV systolic contraction; hypokinesia was defined as a diminished movement or contraction of a segment of the heart muscle.

### Calculation of scar formation in the infarct myocardium by Segment-Medviso software

To determine the accurate amount of scar formation in the myocardial infarct area, the Segment-Medviso software (i.e., MEDVISO company) was utilized in the present study. Briefly, the signal intensity measurement of the scar score was initially obtained at a manually traced irregular region of interest (ROI) of the infract area (white area), followed by Segment-Medviso software analysis.

### Immunohistochemical study

The procedure and protocol for immunohistochemical (IHC) staining were based on our previous report (25–27). Briefly, for IHC staining, rehydrated paraffin sections were first treated with 3% H_2_O_2_ for 30 min and incubated with Immuno-Block reagent (BioSB, Santa Barbara, CA, United States) for 30 min at room temperature. Sections were then incubated with primary antibodies specifically against von Willebrand factor (vWF) (1:400, GR3323044-1, Abcam), Isolectin-B4 (10 μg/mL, ZG0114, Vector Laboratories), cardiac troponin-T (1:100, Gr3263207-2, Abcam), cardiac troponin-I (1:200, GR3248433-4, Abcam), and NM95 (1:200, GR3296957-3, Abcam), while sections incubated with the use of irrelevant antibodies served as controls. Three sections of heart specimens from each pig were analyzed. For quantification, three randomly selected high-power fields (HPFs) (400× for IF studies) were analyzed in each section.

### Histopathological finding of myocardial fibrosis

The procedure and protocol were based on our previous studies (25–27). In detail, hematoxylin and eosin (H&E) and Masson's trichrome staining were utilized for identification of the LV fibrotic area. Three serial sections of LV myocardium in each animal were prepared at 4 µm thickness using Cryostat (Leica CM3050S). The integrated area (µm^2^) of fibrosis on each section was calculated using the Image Tool 3 (IT3) image analysis software (University of Texas, Health Science Center, San Antonio, UTHSCSA; Image Tool for Windows, Version 3.0, United States). Three randomly selected HPFs (100×) were analyzed in each section. After determining the number of pixels in each fibrotic area per HPF, the numbers of pixels obtained from three HPFs were calculated. The procedure was repeated in two other sections of each animal. The mean pixel number per HPF for each animal was then analyzed by summing up all pixel numbers and divided by 9. The mean integrated area (µm^2^) of fibrosis in LV myocardium per HPF was obtained using a conversion factor of 19.24 (since 1 µm^2^ corresponds to 19.24 pixels).

### Western blot analysis

The procedure and protocol for Western blot analysis have been described in our previous reports (25–27). Briefly, equal amounts (50 µg) of protein extracts were loaded and separated by SDS-PAGE using acrylamide gradients. After electrophoresis, the separated proteins were transferred electrophoretically to a polyvinylidene difluoride (PVDF) membrane (Amersham Biosciences). Nonspecific sites were blocked by incubation of the membrane in blocking buffer [5% nonfat dry milk in T-TBS (TBS containing 0.05% Tween 20)] overnight. The membranes were incubated with the indicated primary antibodies [stromal cell-derived growth factor (SDF)-1α] (1:1,000, Cell Signaling), vascular endothelial growth factor (VEGF) (1:1,000, Abcam), p-γH2AX (1:1,000 Cell Signaling), cleaved caspase 3 (1:1,000, Cell Signaling), cleaved Poly (ADP-ribose) polymerase (c-PARP) (1:1,000, Cell Signaling), tumor necrosis factor (TNF)-α (1:1,000, Cell Signaling), interleukin (IL)-1β (1:1,000, Cell Signaling), p-NFκB (1:1,000, Cell Signaling), NOX-1 (1:1,500, Sigma-Aldrich), NOX-2 (1:1,000, Sigma-Aldrich), and oxyblot oxidized protein detection kit purchased from Chemicon (S7150)] for 1 h at room temperature. Horseradish peroxidase-conjugated anti-rabbit immunoglobulin IgG (1:2,000, Cell Signaling) was used as a secondary antibody for 1-h incubation at room temperature. Actin (1:1,000, Millipore) was utilized as internal control. The washing procedure was repeated eight times within 1 h. Immunoreactive bands were visualized by enhanced chemiluminescence (ECL; Amersham Biosciences) and exposed to Biomax L film (Kodak). For quantification, ECL signals were digitized using Labwork software (UVP).

### Laboratory analyses for proving the safety of hBMMSC therapy

To prove that hBMMSCs therapy is safe, we collected the peripheral blood and urine samples prior to and 5 months after AMI induction for hematologic, biochemistry, and urine analyses using laboratory standard methods (refer Tables 1–6).

**Table 1:** Hematological characteristics of three groups at baseline.

Characteristics	Sham (*n* = 3)	AMI-B (*n* = 6)	AMI-M (*n* = 6)	*p-*value
WBC (10^3^/μL)	7.4 ± 0.8	8.5 ± 1.8	9.1 ± 3.0	0.5990
RBC (10^6^/μL)	6.8 ± 0.9	6.0 ± 1.1	6.2 ± 1.0	0.5734
Hb (mg/dL)	12.7 ± 2.0	11.0 ± 2.1	11.7 ± 2.1	0.5537
HCT (%)	38.1 ± 7.8	32.4 ± 8.9	34.6 ± 7.8	0.6519
MCV (fL)	55.6 ± 3.7	53.7 ± 4.4	54.9 ± 4.1	0.8079
MCH (pg/cell)	18.6 ± 0.3	18.4 ± 0.5	18.6 ± 0.7	0.8251
MCHC (g/dL)	33.6 ± 1.6	34.4 ± 2.1	34.0 ± 1.4	0.8211
Platelet (10^3^/μL)	459.0 ± 78.3	431.6 ± 57.1	408.3 ± 83.6	0.6453
Neutrophil (%)	40.8 ± 1.3	43.7 ± 5.0	40.7 ± 3.2	0.4240
Lymphocyte (%)	56.6 ± 2.0	52.9 ± 5.1	55.8 ± 3.1	0.3740
Monocyte (%)	2.3 ± 0.6	2.9 ± 1.1	2.8 ± 0.4	0.5761
Eosinophil (%)	0.2 ± 0.2	0.4 ± 0.3	0.6 ± 0.4	0.2855
Basophil (%)	0.1 ± 0.1	0.1 ± 0.1	0.0 ± 0.0	0.1262

Sham, sham-operated control; AMI-B, STEMI + buffer; AMI-M, AMI + hBMMSCs); AMI, acute myocardial infarction; hBMMSCs, human bone marrow-derived mesenchymal stem cells; WBC, white blood cell; RBC, red blood cell; Hb, hemoglobin; HCT, hematocrit; MCV, mean corpuscular volume; MCH, mean corpuscular hemoglobin; MCHC, mean corpuscular hemoglobin concentration (MCHC).

**Table 2:** Hematological characteristics of three groups at 5 months after AMI induction.

Characteristics	Sham (*n* = 3)	AMI-B (*n* = 6)	AMI-M (*n* = 6)	*p-*value
WBC (10^3^/μL)	9.2 ± 1.6	9.2 ± 2.0	7.9 ± 2.2	0.5465
RBC (10^6^/μL)	6.4 ± 1.2	6.1 ± 0.9	6.2 ± 1.3	0.9368
Hb (mg/dL)	12.9 ± 2.3	12.0 ± 1.5	12.1 ± 1.9	0.7845
HCT (%)	36.8 ± 7.3	33.9 ± 4.3	34.0 ± 5.7	0.7457
MCV (fL)	57.5 ± 3.0	55.6 ± 2.7	55.1 ± 3.9	0.6082
MCH (pg/cell)	20.2 ± 0.6	19.8 ± 0.8	19.7 ± 1.2	0.7671
MCHC (g/dL)	35.3 ± 1.2	35.6 ± 0.5	35.7 ± 0.7	<0.0001
Platelet (10^3^/μL)	410.0 ± 67.7	411.8 ± 54.4	324.2 ± 104.0	0.2135
Neutrophil (%)	41.4 ± 10.0	42.0 ± 6.7	43.2 ± 7.8	0.9462
Lymphocyte (%)	55.6 ± 9.7	54.7 ± 6.2	52.5 ± 8.0	0.9933
Monocyte (%)	2.9 ± 0.5	3.0 ± 0.7	3.6 ± 0.7	0.2830
Eosinophil (%)	0.2 ± 0.2	0.2 ± 0.1	0.4 ± 0.3	0.3194
Basophil (%)	0.0 ± 0.0	0.1 ± 0.1	0.4 ± 0.5	0.2189

Sham, sham-operated control; AMI-B, STEMI + buffer; AMI-M, AMI + hBMMSCs; AMI, acute myocardial infarction; hBMMSCs, human bone marrow-derived mesenchymal stem cells; WBC, white blood cell; RBC, red blood cell; Hb, hemoglobin; HCT, hematocrit; MCV, mean corpuscular volume; MCH, mean corpuscular hemoglobin; MCHC, mean corpuscular hemoglobin concentration (MCHC).

**Table 3:** Biochemistry characteristics of three groups at baseline.

Characteristics	Sham (*n* = 3)	AMI-B (*n* = 6)	AMI-M (*n* = 6)	*p*-value
Aspartate aminotransferase (U/L)	40.7 ± 8.6	45.2 ± 31.9	29.7 ± 4.9	0.5084
Alanine aminotransferase (U/L)	51.0 ± 17.4	55.2 ± 13.4	75.5 ± 41.5	0.4310
Gamma glutamyl transferase (U/L)	50.7 ± 19.3	44.0 ± 6.4	45.7 ± 10.7	0.7365
Alkaline phosphatase (U/L)	144.3 ± 18.8	121.8 ± 47.5	119.7 ± 30.2	0.6310
Blood urea nitrogen (mg/dL)	4.0 ± 0.0	5.8 ± 1.6	7.8 ± 4.7	0.2855
T-bilirubin (mg/dL)	0.1 ± 0.0	0.2 ± 0.3	0.1 ± 0.0	0.6636
Creatinine (mg/L)	1.0 ± 0.2	0.7 ± 0.1	0.8 ± 0.1	0.0271*
Total cholesterol (mg/L)	61.3 ± 1.5	49.5 ± 7.0	47.8 ± 7.4	0.0407*
Triglyceride (mg/L)	31.3 ± 12.7	30.8 ± 17.3	26.7 ± 17.9	0.9041
Fasting plasma glucose (mg/dL)	98.0 ± 21.6	68.3 ± 11.5	84.2 ± 9.1	0.0345
Calcium level (mg/L)	9.4 ± 0.4	9.4 ± 0.1	9.4 ± 0.4	0.4019
Phosphorus (mmol/L)	6.9 ± 1.1	6.9 ± 0.1	6.8 ± 0.4	0.9518
Sodium (mmol/L)	143.0 ± 2.6	142.7 ± 3.1	144.7 ± 2.7	0.5258
Potassium (mmol/L)	4.1 ± 0.9	5.0 ± 1.4	5.1 ± 0.4	0.3876
Chloride (mmol/L)	104.7 ± 2.5	104.2 ± 3.7	106.3 ± 0.8	0.4602
Total protein (g/dL)	7.1 ± 0.4	6.9 ± 0.3	6.8 ± 0.3	0.4706
Albumin (g/dL)	4.3 ± 0.4	3.8 ± 0.2	3.8 ± 0.3	0.0732
Globulin (g/dL)	2.8 ± 0.3	3.1 ± 0.2	3.0 ± 0.2	0.2328
Amylase (U/L)	416 ± 185	688 ± 216	731 ± 102	0.0741
Creatine kinase (U/L)	303 ± 69	383 ± 110	315 ± 115	0.5019

Sham, sham-operated control; AMI-B, STEMI + buffer; AMI-M, AMI + hBMMSCs; AMI, acute myocardial infarction; hBMMSCs, human bone marrow-derived mesenchymal stem cells.

*Indicated *p* < 0.05.

**Table 4:** Biochemistry characteristics of three groups at 5 months after AMI induction.

Characteristics	Sham (*n* = 3)	AMI-B (*n* = 6)	AMI-M (*n* = 6)	*p*-value
Aspartate aminotransferase (U/L)	27.0 ± 3.6	28.5 ± 5.8	33.7 ± 10.1	0.4241
Alanine aminotransferase (U/L)	51.0 ± 15.9	51.2 ± 17.9	55.8 ± 9.0	0.8547
Gamma glutamyl transferase (U/L)	36.7 ± 5.5	36.7 ± 5.6	44.7 ± 7.0	0.1251
Alkaline phosphatase (U/L)	108.3 ± 27.5	123.5 ± 16.2	113.8 ± 38.8	0.7608
Blood urea nitrogen (mg/dL)	7.3 ± 2.9	9.3 ± 2.9	8.5 ± 2.3	0.6075
Total bilirubin (mg/dL)	0.1 ± 0.0	0.1 ± 0.0	0.1 ± 0.0	>0.9999
Creatinine (mg/L)	0.8 ± 0.2	0.9 ± 0.1	0.8 ± 0.1	0.4150
Total cholesterol (mg/L)	57.7 ± 16.0	44.0 ± 16.4	41.2 ± 15.6	0.3807
Triglyceride (mg/L)	22.7 ± 11.4	28.5 ± 13.8	21.2 ± 7.4	0.5777
Fasting plasma glucose (mg/dL)	77.0 ± 6.0	77.3 ± 17.5	73.3 ± 9.9	0.8726
Calcium (mg/L)	9.1 ± 0.1	9.3 ± 0.5	9.1 ± 0.3	0.6524
Phosphorus (mmol/L)	6.4 ± 0.5	5.9 ± 0.2	6.1 ± 0.5	0.2880
Sodium (mmol/L)	138.7 ± 9.7	144.0 ± 5.1	142.5 ± 2.3	0.4553
Potassium (mmol/L)	4.0 ± 0.2	3.9 ± 0.3	4.1 ± 0.3	0.5549
Chloride (mmol/L)	100.3 ± 9.0	105.0 ± 4.7	103.2 ± 1.9	0.4835
Total protein (g/dL)	7.2 ± 0.2	7.3 ± 0.5	7.2 ± 0.6	0.9411
Albumin (g/dL)	3.9 ± 0.2	3.8 ± 0.3	3.9 ± 0.4	0.8690
Globulin (g/dL)	3.2 ± 0.4	3.5 ± 0.4	3.3 ± 0.2	0.4528
Amylase (U/L)	401 ± 195	709 ± 233	748 ± 156	0.0881
Creatine kinase (U/L)	283 ± 157	217 ± 77	435 ± 328	0.3334

Sham, sham-operated control; AMI-B, STEMI + buffer; AMI-M, AMI + hBMMSCs; AMI, acute myocardial infarction; hBMMSCs, human bone marrow-derived mesenchymal stem cells.

**Table 5:** Urine examination for three groups at baseline.

Characteristics	Sham (*n* = 3)	AMI-B (*n* = 6)	AMI-M (*n* = 6)	*p-*value
Specific gravity	1.01 ± 0.01	1.01 ± 0.01	1.02 ± 0.01	0.5742
pH	8.0 ± 0.5	7.3 ± 0.8	7.8 ± 0.6	0.3375
Glucose urine	Negative	Negative	Negative	
Protein	Negative	Negative	Negative	
Occult blood	Negative	Negative	Negative	
Urobilinogen, EU/dL	0.2	0.2	0.2	>0.9999
Bilirubin	Negative	Negative	Negative	
Ketone	Negative	Negative	Negative	
Nitrite	Negative	Negative	Negative	
Leukocytes	Negative	Negative	Negative	

Sham, sham-operated control; AMI-B, STEMI + buffer; AMI-M, AMI + hBMMSCs; AMI, acute myocardial infarction.

**Table 6:** Urine examination for three groups at 5 months after AMI induction.

Characteristics	Sham (*n* = 3)	AMI-B (*n* = 6)	AMI-M (*n* = 6)	*p-*value
Specific gravity	1.02 ± 0.01	1.02 ± 0.01	1.02 ± 0.01	0.4420
pH	7.0 ± 0.0	6.5 ± 2.5	7.4 ± 1.0	0.5765
Glucose urine	Negative	Negative	Negative	
Protein	Negative	Negative	Negative	
Occult blood	Negative	Negative	Negative	
Urobilinogen, EU/dL	0.2	0.2	0.2	>0.9999
Bilirubin	Negative	Negative	Negative	
Ketone	Negative	Negative	Negative	
Nitrite	Negative	Negative	Negative	
Leukocytes	Negative	Negative	Negative	

Sham (group 1), sham-operated control; AMI-B (group 2), AMI + buffer; AMI-M (group 3), AMI + hBMMSC; AMI, acute myocardial infarction; hBMMSCs, human bone marrow-derived mesenchymal stem cells.

All scale bars in lower right corner represent 50 µm.

### Statistical analysis

Quantitative data are expressed as mean ± SD. Statistical analyses were performed using SAS statistical software for Windows Version 8.2 (SAS Institute, Cary, NC, United States). One-way ANOVA was conducted, followed by Bonferroni multiple comparison *post-hoc test* for comparing variables among groups. A probability value <0.05 was considered statistically significant.

## Results

### Serial changes of LVEF, ST-segment resolution, and the body weight among the three groups of the animals

To elucidate the therapeutic impact of early intracoronary administration of hBMMSCs (i.e., the product OmniMSC) on salvaging the LV function in porcine AMI, serial transthoracic echocardiography was performed in the present study. The result demonstrated that the baseline LVEF did not differ among the three groups. However, by 1.5 h and 3 h as well as by 7 h after AMI induction, the LVEF was significantly lower in groups 2 (AMI + buffer) and 3 (AMI + hBMMSCs), but it showed no difference between the latter two groups. By 1, 2, 3, 4, and 5 months after AMI induction, LVEF was still significantly lower in groups 2 and 3 than that of group 1. However, this parameter was significantly higher in group 3 than in group 2 by these time points after AMI induction, suggesting that early administration of this xenogeneic MSC (i.e., hBMMSCs) therapy effectively salvaged the porcine LV function in the setting of AMI.

It is well known that the ST-segment resolution is a crucial predictor of IRA patency and effectiveness of microcirculatory perfusion. As we expected, serial ECG examinations showed that after 8 h LAD occlusion, the ST-segment resolution was remarkably greater in group 3 than in group 2. This finding, once again, suggested that early and prompt intracoronary administration of this hBMMSC therapy effectively rescued the LV myocardium and reduced the LV infarct area in porcine AMI.

By time point of baseline and at 1, 2, and 4 weeks after AMI induction, the body weight (BW) was similar among the three groups. However, by 2, 4, and 5 months after AMI induction, this parameter was significantly higher in group 1 than groups 2 and 3, but it did not differ among groups 2 and 3. This finding might imply that AMI would attenuate the growth percentile in the animals.

### Time courses of cardiac MRI evaluations for identification of cardiac motion and scar score

To accurately estimate the abnormal motion of the infarct region and scar score, cardiac MRI was utilized in the present study. The result of this study demonstrated that 2 months after AMI induction, the abnormal cardiac muscle exercise score and scar score in the infarct zone were significantly higher in groups 2 and 3 than in group 1, but they did not show significant difference between groups 2 and 3. Additionally, even at the end of the study period, these parameters were still significantly abnormally high in groups 2 and 3 and also notably higher in group 2 than in group 3. Our finding from the cardiac MRI study precisely demonstrated that early intracoronary administration of these xenogeneic MSCs effectively preserved LV function and reduced infarct size and LV remodeling in porcine AMI.

### MMT assay 5 months after AMI induction

To assess the pathologically anatomical macrostructure of infract region, the MMT assay was conducted in the present study. The result showed that the infarct area at the papillary muscle level was significantly higher in group 2 than in groups 1 and 3 and significantly higher in group 3 than in group 1. Additionally, the summation of total infarct mass volume displayed an identical pattern of infarct area among the groups. Our findings, once again, proved that the early administration of xenogeneic MSCs effectively attenuated the LV infarct size in AMI animals.

### Histopathological findings of LV myocardium 5 months after AMI induction

Histopathological assessment, i.e., cellular level of investigation, was another tool in the present study utilized for measuring the impact of hBMMSCs therapy on salvaging the LV myocardium. The microscopic finding of H&E stain demonstrated that the infarct area was significantly higher in group 2 than in group 1 that was significantly reversed in group 3 animals after receiving hBMMSC therapy. Additionally, Masson's trichrome stain demonstrated that the fibrotic area of LV myocardium was significantly higher in group 2 than in groups 1 and 3 and significantly higher in group 3 than in group 1. These findings were compatible with the findings of cardiac MRI and implied that early administration of xenogeneic MSCs notably ameliorated the LV myocardial fibrosis.

### Immunohistochemical staining for identification of troponin-T and troponin-I expressions of LV infarct myocardium 5 months after AMI induction

Myocardial tissues of troponin-T and troponin-I are two cardiomyocyte-specific proteins that play key roles in regulating myocardial contraction. Thus, loss of their expressions in LV myocardium always indicates myocardial damage. To elucidate whether the myocardium was effectively preserved by hBMMSC therapy, IHC stain was utilized in this study. The result demonstrated that troponin-T and troponin-I expressions were significantly higher in group 1 than in groups 2 and 3 and significantly higher in group 3 than in group 2. Our finding once again supported that early administration of xenogeneic MSCs remarkably salvaged myocardial injury in setting of AMI.

### Endothelial cell marker, vascular niche, and protein expressions of angiogenesis biomarkers in infarcted LV myocardium 5 months after AMI induction

To assess the expression of vWF+ cells in the vessels of infarct area, as a biomarker of endothelial cells (ECs) that reflected the integrity of ECs, IHC stain was performed in harvested infarcted LV myocardium. The result demonstrated that this parameter was significantly higher in group 1 than in groups 2 and 3 and significantly higher in group 3 than in group 2. On the other hand, the cellular expression of isolectin-B4, an indicator of vascular niche (i.e., a vasculature-density marker), was significantly higher in group 2 than in groups 1 and 3, and significantly higher in group 3 than in group 1, implicating an intrinsic response to ischemic stimulation that was partially attenuated by MSC treatment.

To elucidate whether the hBMMSCs treatment would upregulate the angiogenesis biomarkers in the infarcted LV myocardium, Western blot was utilized in the present study. The result demonstrated that the protein expressions of SDF-1α and VEGF were significantly and progressively increased from groups 1 to 3, implicating an intrinsic response to ischemic stimulation that was augmented by hBMMSCs, suggesting that this cell therapy would enhance the expressions of angiogenic factors and angiogenesis for restoring blood flow in the ischemic zone.

### Protein expressions of inflammation, oxidative stress, and apoptosis/DNA damage in infarcted LV myocardium 5 months after AMI induction

It is well known that AMI always elicits inflammation, oxidative stress, and cell death. Thus, Western blot was used for the evaluation of these aforementioned molecular perturbations. The result demonstrated that the protein expressions of IL-1β, TNF-α, and NF-κB, three indices of inflammation, and protein expressions of NOX-1, NOX-2, and oxidized protein, three indicators of oxidative stress, were significantly increased in group 2 than in groups 1 and 3, and significantly increased in group 3 than in group 1. Additionally, the protein expressions of cleaved caspase 3 and cleaved PARP, two indices of apoptosis, and protein expression of γ-H2AX, an indicator of DNA-damaged biomarker, exhibited an identical pattern of inflammation among the three groups. Our findings established that early administration of xenogeneic MSCs notably suppressed inflammation and oxidative stress in infarcted LV myocardium.

### Supplementary parameters, including laboratory analyses, for proving the safety of hBMMSC therapy

To prove that the hBMMSC therapy was safe, we collected the peripheral blood and urine samples prior to and 5 months after AMI induction for hematologic, biochemistry, and urine analyses using laboratory standard methods (refer Tables 1–6). The majority of the parameters including the hematologic parameters, biochemistry parameters, and urine parameters at the time points of baseline and 5 months after AMI induction did not differ among the three groups.

### Characteristics of hBMMSCs

hBMMSCs were expanded from ITRI's GTP-compliant MSC banks and characterized by the morphology, plastic adherent, CD marker expression, and trilineage differentiation property. hBMMSCs could differentiate into adipocytes, osteoblasts, and chondroblasts in the induction medium (Supplementary Figure S1A). hBMMSCs express CD105, CD73, and CD90 and lack flow cytometric analysis to clarify the high population of hBMMSCs markers in our OmniMSC-AMI product (Supplementary Figure S1B). Finally, we also proved that our MSCs could effectively suppress the proliferation of T-cells (Supplementary Figure S1C).

To prove that the ability of frozen hBMMSCs was noninferior to fresh hBMMSCs for suppressing the proliferation of T-cells, these cells were co-cultured with T-cells and the result showed that the suppression of T-cell proliferation was similar among these MSCs (Supplementary Figure S1A).

To prove that the ability of frozen hBMMSCs was noninferior to fresh hBMMSCs for suppressing the inflammation of the peripheral blood mononuclear cells (PBMCs), these MSCs were co-cultured with PBMCs. The result showed the inflammatory cytokines, including TNF-α (Supplementary Figure S1B) and IFN-γ (Supplementary Figure S1C), were remarkably and equally suppressed by those of fresh and frozen MSCs. On the other hand, the cell viability of these MSCs did not differ in fresh and frozen conditions (Supplementary Figure S2).

## Discussion

This study examined the safety and also attempted to explore the therapeutic impact of xenogeneic hBMMSCs (i.e., product name: OmniMSC) on rescuing the LV myocardium and its function in big AMI animals delivered several striking preclinical implications. Frist, intracoronary administration of xenogeneic hBMMSC therapy for AMI porcine was safe. Second, early intracoronary administration of hBMMSCs after AMI induction in porcine effectively preserved LV performance. Third, this preservation of LVEF was mainly through salvaging LV myocardium, resulting in the attenuation of inflammation and oxidative stress generation, as a subsequence in reducing the infarct myocardial mass.

The main mission of this study was to clarify whether the xenogeneic MSCs treatment, i.e., hBMMSCs therapy for porcine AMI, was really safe that would truly affect the upcoming phase I clinical trial entitled “Intracoronary administration of OmniMSC-AMI for acute STEMI patients undergoing primary PCI—phase I clinical trial” to assess the safety. In this study, we carefully evaluated a vast number of parameters, including (1) vital signs (i.e., temperature, blood pressure, heart rate, activity, and appetite after AMI induction), (2) mortality rate, (3) serial measures of body weight after AMI induction, and (4) serial urine examination and serial blood samples for biochemistries, i.e., hematologic, liver, and renal function as well as myocardial damaged biomarkers after hBMMSC administration. All the parameters were repeatedly discussed and challenged by experts of IRB committees, followed by our defense and explanation in expert meetings, leading to the final conclusion of the experts’ comments to unanimously agree that our preclinical trial was safe. Thus, our phase I clinical trial was permitted to be performed soon and will start in February 2023.

In this study, we attempted to explore the second mission, i.e., to elucidate the “efficacy” of OmniMSC therapy for porcine AMI. One important finding in the present study was that compared with the Sham group, LVEF was persistently reduced in the AMI-B group since receiving AMI-induced procedure up to the end of study period (i.e., by the end of 5 months). Of interest was that serial measurement of the LVEF showed no improvement in AMI-B after AMI induction, suggesting that the buffer treatment did not offer any benefit for improving the LV function.

**Figure 1:**
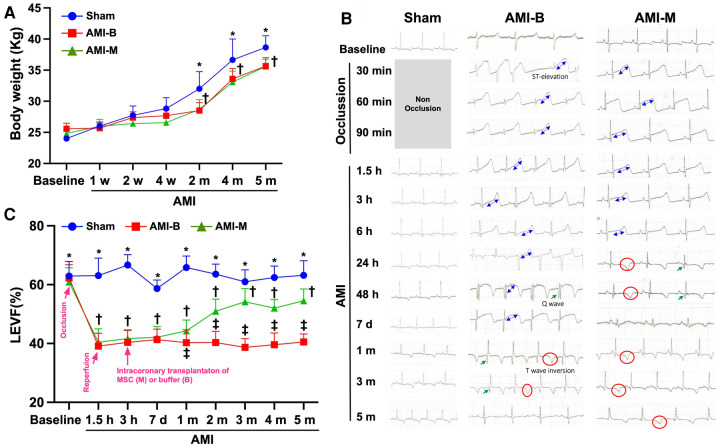
Serial changes of LVEF, ST-segment resolution, and the BW among the three groups of the animals. (**A**) The body weight at baseline to 4 weeks after AMI induction did not differ among the three groups, *p* > 0.5. However, the body weight was notably increased in Sham than in AMI-B and AMI-M groups, * vs. †, *p* < 0.01. (**B**) Illustrating the serial ECG examinations and the results showed that after 8 h AMI induction, the ST-segment resolution was remarkably greater in AMI-M (i.e., group 3) than in AMI-B (i.e., group 2). (**C**) By 1.5 h and 3 h as well as 7 h after AMI induction, the LVEF was significantly lower in groups 2 and 3 than group 1 (Sham), * vs. †, *p* < 0.0001. Additionally, 1, 2, 3, 4, and 5 months after AMI induction, LVEF was still significantly lower in groups 2 and 3 than that in group 1, * vs. other groups with different symbols (†, ‡), *p* < 0.0001. All statistical analyses were performed by one-way ANOVA, followed by Bonferroni multiple comparison *post-hoc test*. Sham (group 1), sham-operated control; AMI-B (group 2), AMI + buffer; AMI-M (group 3), AMI + hBMMSC; AMI, acute myocardial infarction; hBMMSCs, human bone marrow-derived mesenchymal stem cells; LVEF, left ventricular ejection fraction; BW, body weight; ECG, electrocardiogram.

The most important finding in the present study was that as compared with the AMI-B group, LVEF was notably improved at the early time point and even much more increased in the AMI-M group after AMI induction (refer Figure 1). Intriguingly, when we looked at the time course of measuring this LVEF, we even discovered that the remarkable improvement of this parameter as early as 1 week after hBMMSC therapy (refer Figure 1). Interestingly, our previous study has demonstrated that early (i.e., 3 h after AMI induction by LAD ligation) direct implantation of autologous bone marrow-derived MSCs into the LV myocardium of mini-pigs significantly improved the LVEF (25). Our previous clinical study has also demonstrated that shortening the time span between chest pain onset and reperfusion time (i.e., total ischemic time) to less than 4.0 h was crucial in reducing myocardial necrosis and improving LV function and 30-day adverse clinical event (42). Accordingly, the result of the present study by utilizing the xenogeneic rather than by the autologous MSCs corroborated with the finding of our previous study (25, 42) highlighting that early administration of MSCs may be an innovative treatment for salvaging more dying myocardium in AMI patients just after primary PCI.

**Figure 2:**
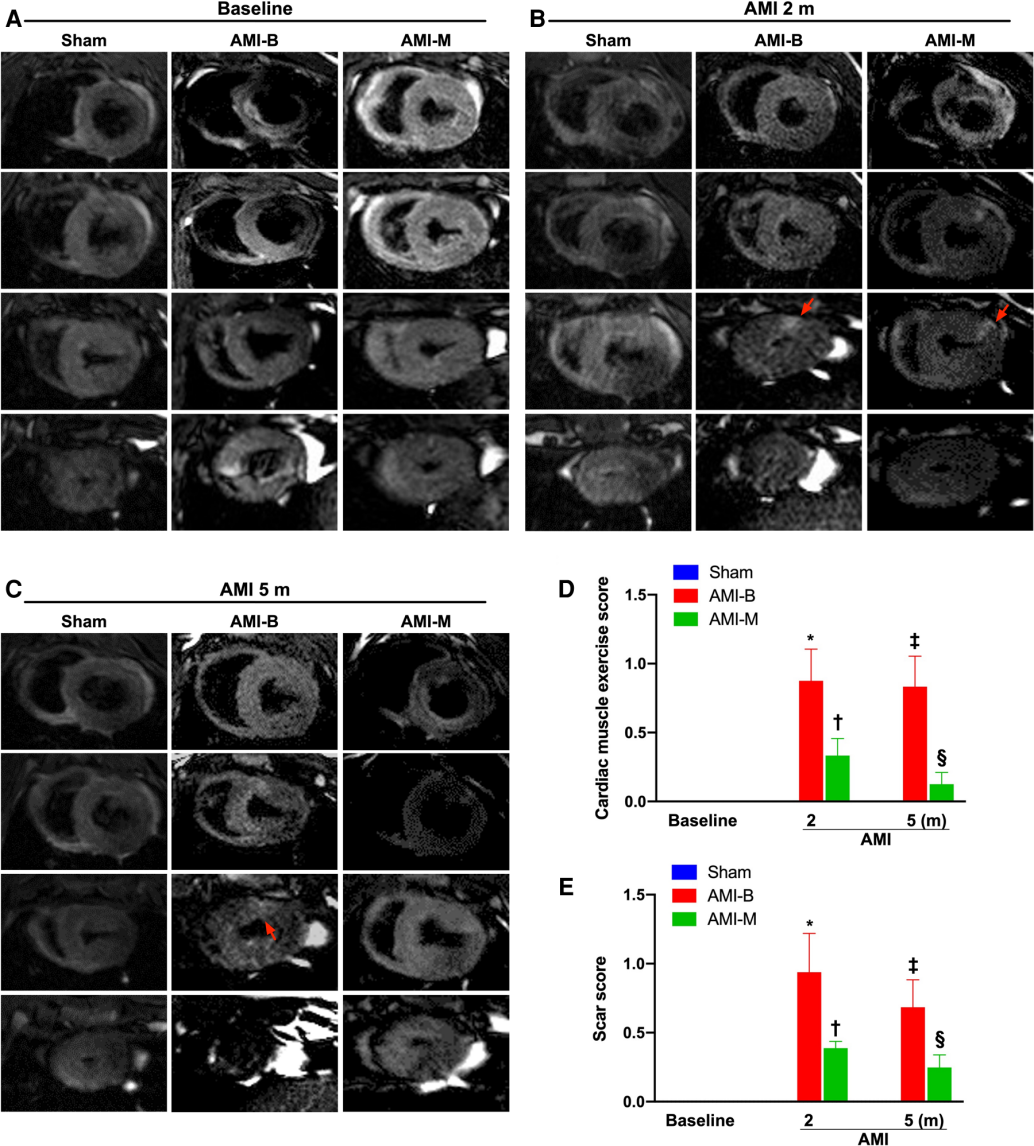
Time courses of cardiac MRI evaluations for identification of abnormal cardiac muscle exercise score and scar score. (**A–C**) The cardiac MRI finding for identification of cardiac muscle exercise score and scar score at baseline (**A**), 2 months after AMI induction (**B**), and 5 months after AMI induction (**C**), respectively. The red arrows indicate myocardial scars. (**D**) Analytical result of abnormal cardiac muscle exercise score 2 months after AMI induction, * vs. †, *p* < 0.001; analytical result of abnormal cardiac muscle exercise score 5 months after AMI induction, ‡ vs. §, *p* < 0.0001. (**E**) Analytical result of myocardial scar score 2 months after AMI induction, * vs. †, *p* < 0.001; analytical result of myocardial scar score 5 months after AMI induction, ‡ vs. §, *p* < 0.0001. Sham (group 1), sham-operated control; AMI-B (group 2), AMI + buffer; AMI-M (group 3), AMI + hBMMSC; AMI, acute myocardial infarction; hBMMSCs, human bone marrow-derived mesenchymal stem cells; MRI, magnetic resonance imaging.

**Figure 3:**
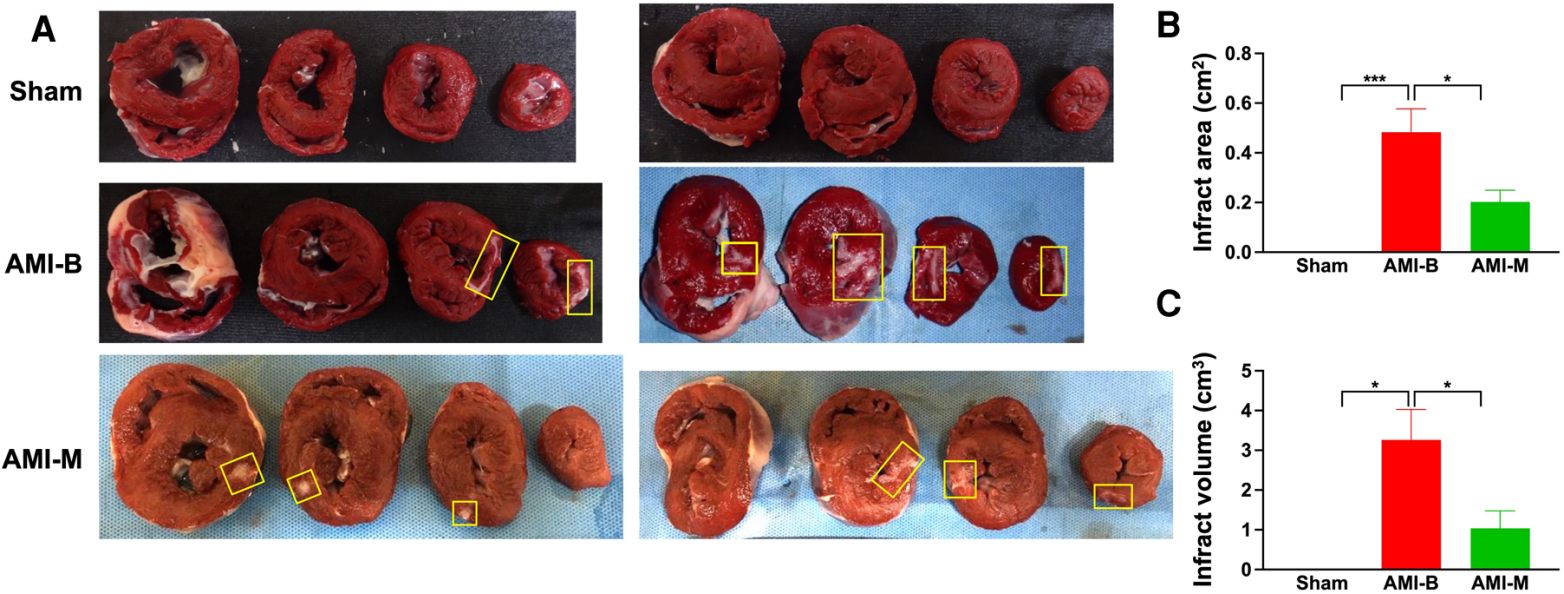
MMT assay for assessing the infarct area and infarct volume 5 months after AMI induction. (**A**) The cross-section of MTT assay for identifications of infarct area (i.e., at papillary muscle level). Yellow square boxes indicated the infarct area. (**B**) Analytical result of infarct area, * indicated *p* < 0.01; ***p < 0.0001. (**C**) *all *p* < 0.01. Sham (group 1), sham-operated control; AMI-B (group 2), AMI + buffer; AMI-M (group 3), AMI + hBMMSC; AMI, acute myocardial infarction; hBMMSCs, human bone marrow-derived mesenchymal stem cells.

**Figure 4:**
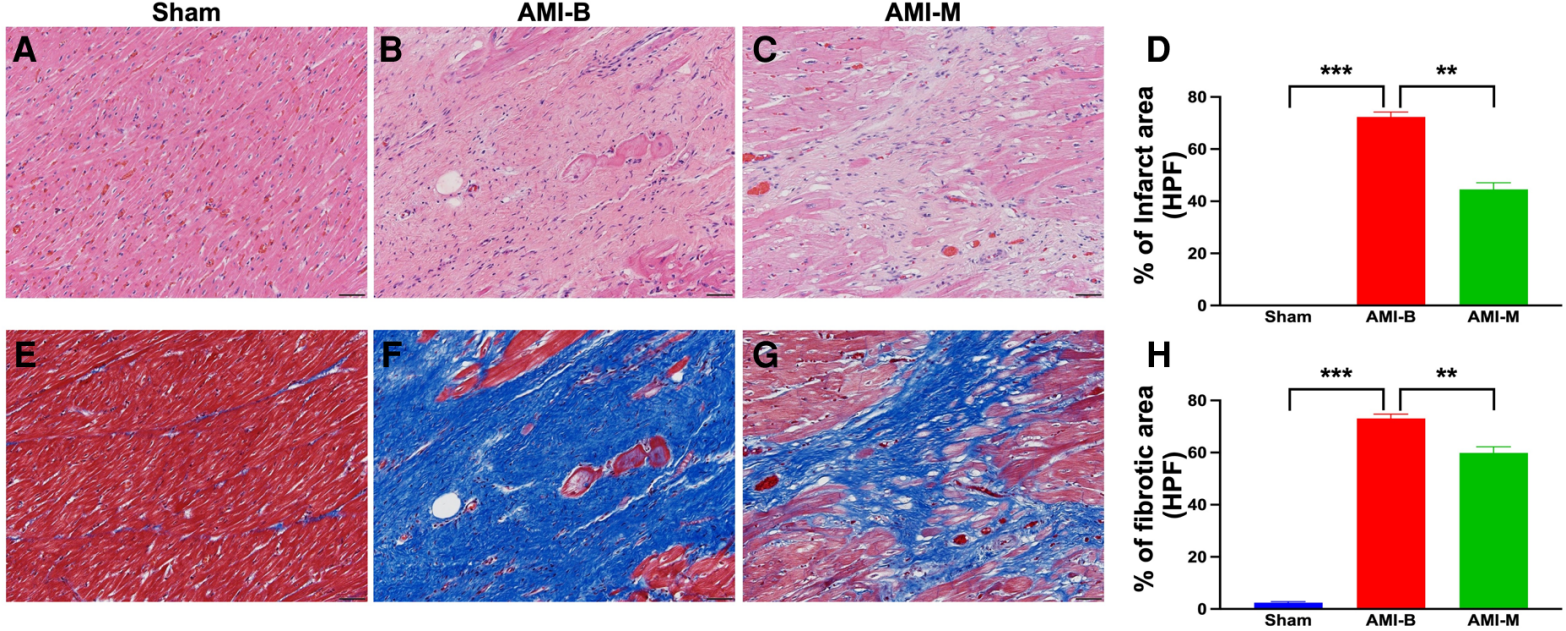
Histopathological assessment of LV myocardium 5 months after AMI induction. (**A–C**) The microscopic finding (200×) of H&E stain for identification of infarct area (red-white area). (**D)** Analytical result of the infarct area (%)/HPF; **p < 0.001; ***p < 0.00.1. (**E–G)** The microscopic finding (200×) of Masson's trichrome stain for identification of the fibrotic area (blue color). (**H)** Analytical result of the fibrotic area (%)/per HPF); **p < 0.001; ***p < 0.0001. All scale bars in lower right corner represent 50 µm. Sham (group 1), sham-operated control; AMI-B (group 2), AMI + buffer; AMI-M (group 3), AMI + hBMMSC; AMI, acute myocardial infarction; hBMMSCs, human bone marrow-derived mesenchymal stem cells; LV, left ventricular; HPF, high-power field; H&E, hematoxylin and eosin.

**Figure 5:**
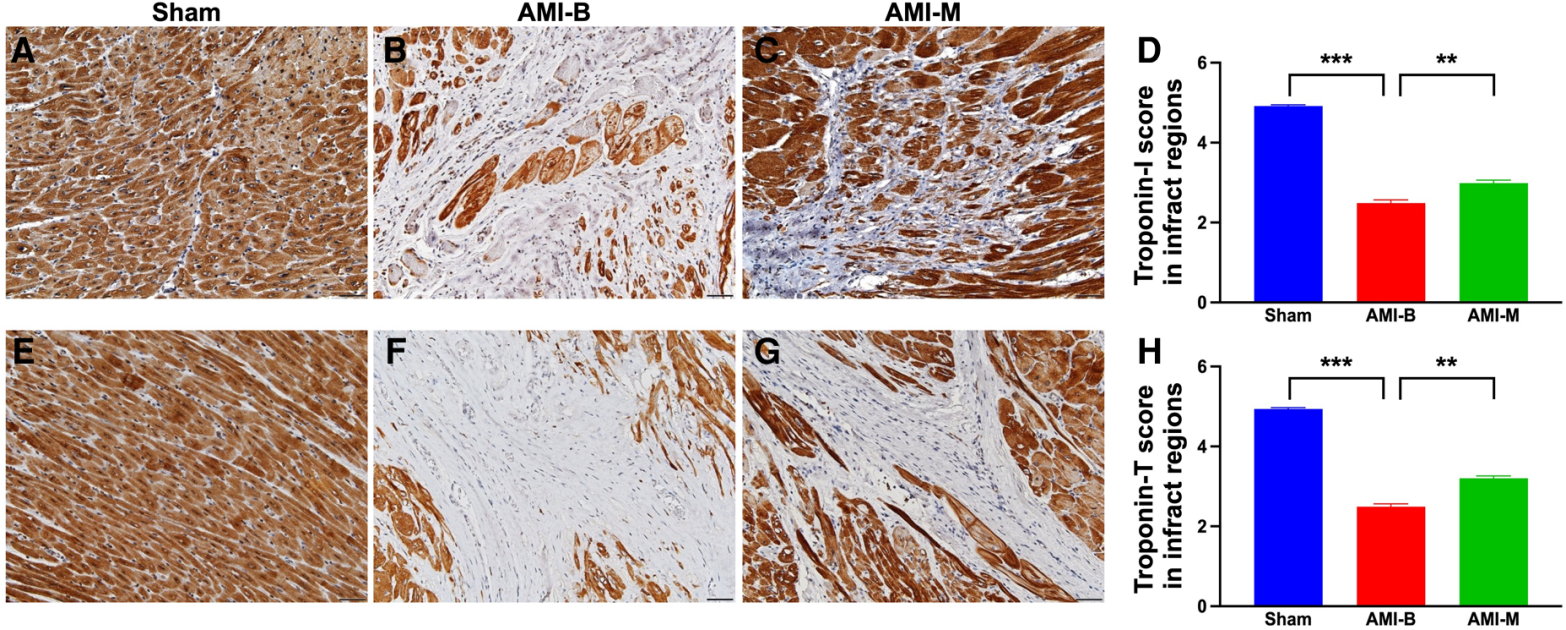
Immunohistochemical staining for identification of troponin-I and troponin-T expressions of LV infarct myocardium by 5 months after AMI induction. (**A–C**) The microscopic finding (200×) of IHC stain for identification of the cellular expression of troponin-I (gray color). (**D**) Analytical result of the expression of IHC-stained intensity score of troponin-I; ***p < 0.0001; **p < 0.001. (**E–G**) The microscopic finding (200×) of IHC stain for identification of the cellular expression of troponin-T (gray color). (**H**) Analytical result of the expression of IHC-stained intensity score of troponin-T; ***p < 0.0001; **p < 0.001. All scale bars in lower right corner represent 50 µm. Sham (group 1), sham-operated control; AMI-B (group 2), AMI + buffer; AMI-M (group 3), AMI + hBMMSC; AMI, acute myocardial infarction; hBMMSCs, human bone marrow-derived mesenchymal stem cells; LV, left ventricular; IHC, immunohistochemical.

Cardiac MRI has been universally agreed that it is a really reliable noninvasive imaging tool for accurately evaluating the cardiac function (41, 43). To mimic the application of cardiac MRI for assessment of heart function in AMI patients, serial measurements (i.e., at baseline and 2 and 5 months after AMI) of abnormal cardiac muscle exercise score and scar score in the infarct region in three groups of the animals were conducted in the present study. An essential finding in the present study was that the abnormal cardiac muscle exercise score (defined as summarized scores of dyskinesia, akinesia, and hypokinesis) was lowest in the Sham group and significantly lower in AMI-M than in AMI-B animals (refer Figure 2). The cardiac MRI finding, therefore, was a mirror image of transthoracic echocardiography, indicating that hBMMSCs treatment would be an innovative weapon for the treatment of AMI. On the other hand, the cardiac MRI study demonstrated that the cardiac scar score was significantly higher in AMI-B animals than in AMI-M animals (refer Figure 2). Consistently, MTT assay for the pathologically anatomical examination demonstrated both the infarct area and infarct volume of LV myocardium displayed an identical cardiac scar score between the two groups (refer Figure 3). Moreover, immunohistochemical staining revealed that the infarct/fibrotic areas of LV myocardium also exhibited an identical pattern (refer Figure 4), whereas the expression of troponin-I/troponin-T in the infarct area (refer Figure 5) exhibited an opposite pattern of the cardiac scar score. These findings, mutually supporting each other, could explain why the LV performance was substantially preserved in AMI-M animals than that of AMI-B animals.

**Figure 6:**
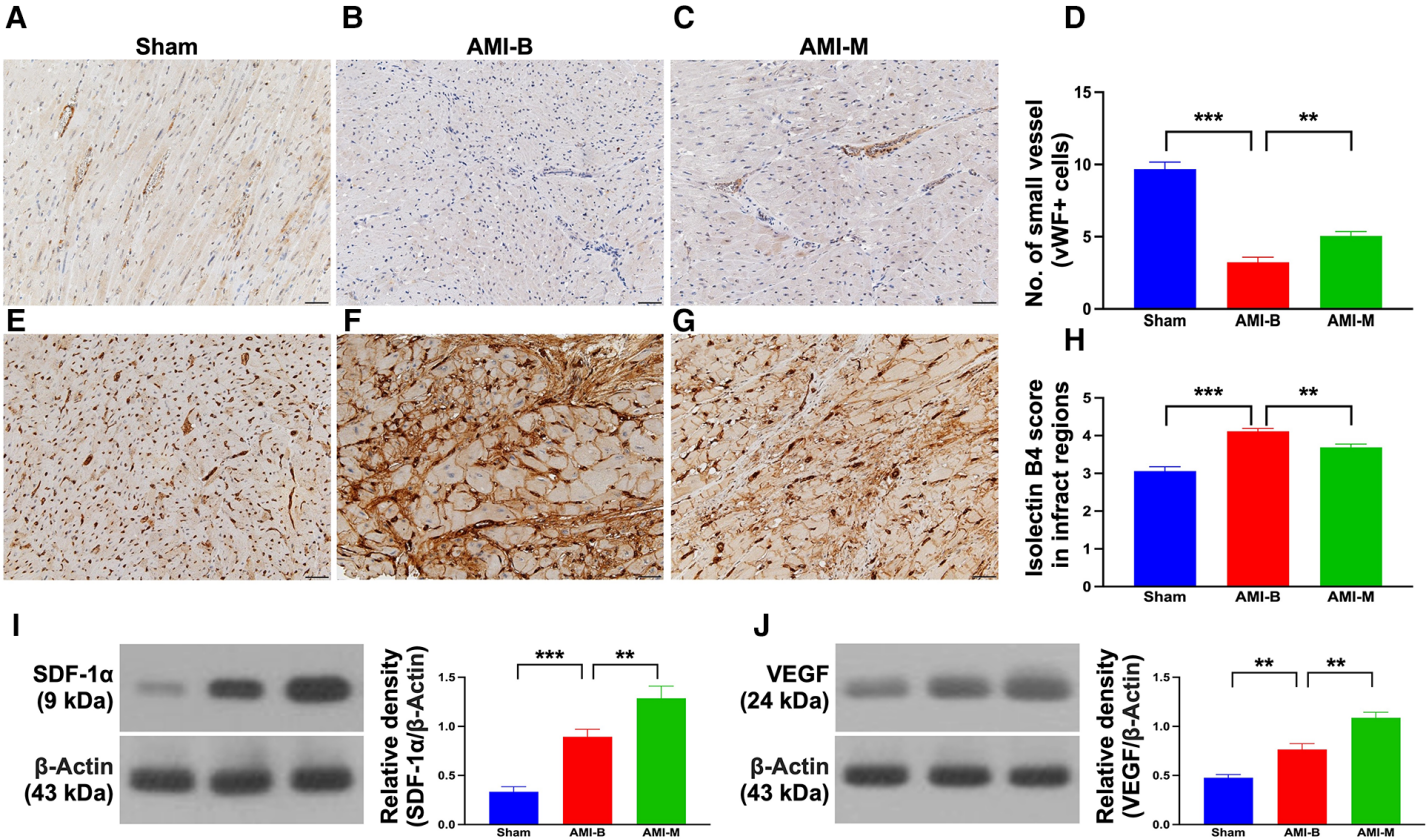
Endothelial cell marker, vascular niche, and protein expressions of angiogenesis biomarkers in LV infarct myocardium 5 months after AMI induction. (**A–C**) The microscopic finding (200×) of IHC stain for identification of cellular expression of vWF in the small vessels (gray color). (**D**) Analytical result of number of small vessels with positively-stained vWF; ***p < 0.0001; **p < 0.001. (**E–G**) The microscopic finding (200×) of IHC stain for identification of cellular expression of isolectin-B4 in infarct myocardium (gray color). (**H**) Analytical result expression of IHC-stained intensity score of isolectin-B4; ***p < 0.0001; **p < 0.001. All scale bars in lower right corner represent 50 µm. (**I**) Protein expression of SDF-1*α*; ***p < 0.0001; **p < 0.001. (**J**) Protein expression of VEGF; ***p < 0.0001; **p < 0.001. Sham (group 1), sham-operated control; AMI-B (group 2), AMI + buffer; AMI-M (group 3), AMI + hBMMSC; AMI, acute myocardial infarction; hBMMSCs, human bone marrow-derived mesenchymal stem cells; LV, left ventricular; IHC, immunohistochemical; vWF, von Willebrand factor; SDF, stromal cell-derived factor; VEGF, vascular endothelial growth factor.

**Figure 7:**
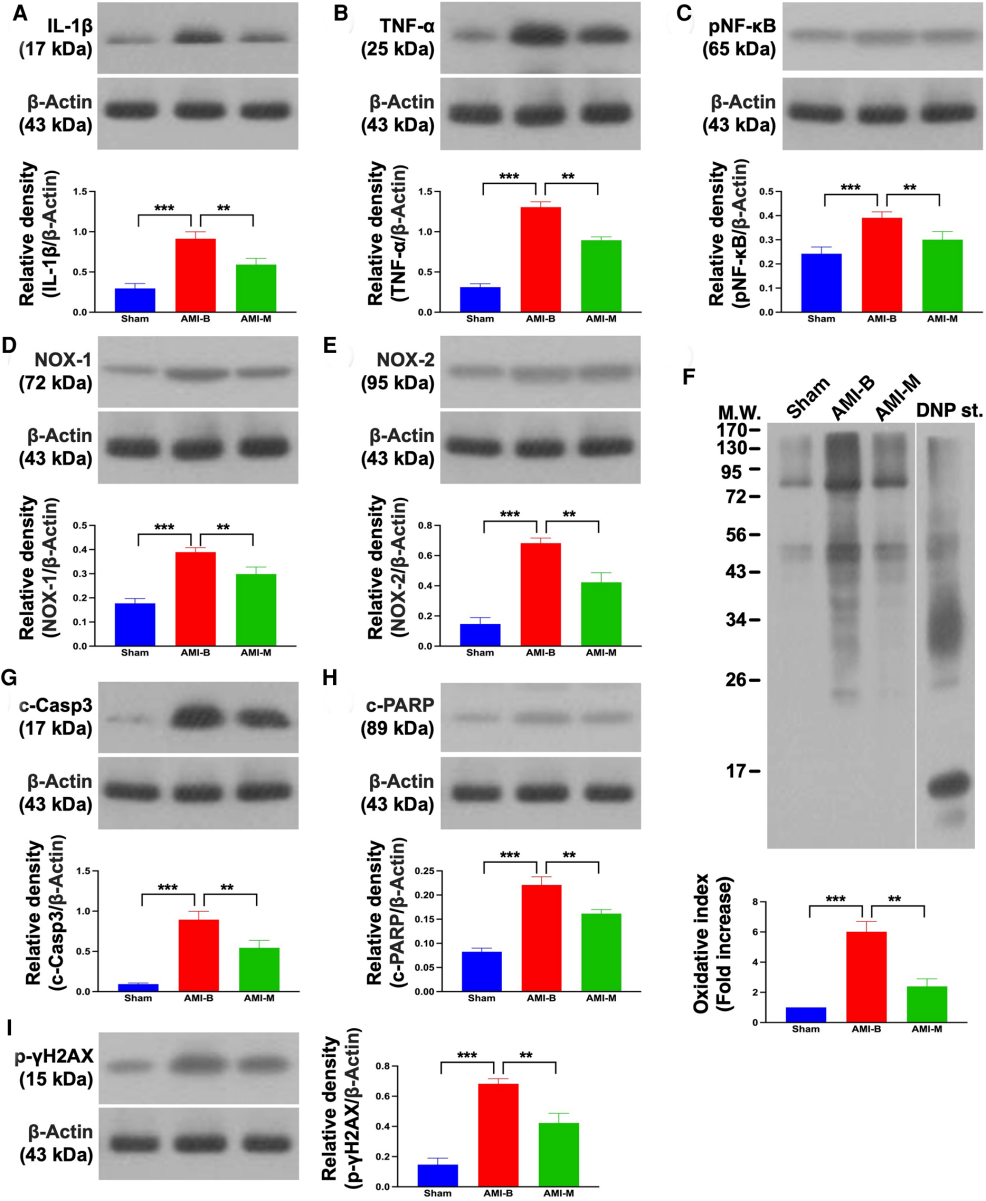
Protein expressions of inflammation, oxidative stress, and apoptosis/DNA damage in LV infarct myocardium 5 months after AMI induction. (**A**) Protein expression of IL-1β; ***p < 0.0001; **p < 0.001. (**B**) Protein expression of TNF-*α*; ***p < 0.0001; **p < 0.001. (**C**) Protein expression of NF-κB; ***p < 0.0001; **p < 0.001. (**D**) Protein expression of NOX-1; ***p < 0.0001; **p < 0.001. (**E**) Protein expression of NOX-2; ***p < 0.0001; **p < 0.001. (**F**) Oxidized protein expression; ***p < 0.0001; **p < 0.001 (Note: the left and right lanes shown on the upper panel represent protein molecular weight marker and control oxidized molecular protein standard, respectively). (**G**) Protein expression of cleaved caspase 3 (c-Casp3); ***p < 0.0001; **p < 0.001. (**H**) Protein expression of c-PARP; ***p < 0.0001; **p < 0.001. (**I**) Protein expression of γ-H2AX; ***p < 0.0001; **p < 0.001. Sham (group 1), sham-operated control; AMI-B (group 2), AMI + buffer; AMI-M (group 3), AMI + hBMMSC; AMI, acute myocardial infarction; hBMMSCs, human bone marrow-derived mesenchymal stem cells; LV, left ventricular; IL, interleukin; TNF, tumor necrosis factor; NF, nuclear factor; MW, molecular weight; DNP, 1-3 dinitrophenylh ydrazone.

The readers would be much more interested in what happened in the cellular–molecular levels of LV myocardium after this MSC treatment. An essential finding in the present study was that as compared with the Sham group, inflammation, oxidative stress, and apoptosis as well as the DNA-damaged biomarkers were substantially increased in the AMI-B group. Interestingly, previous clinical and experimental studies (17, 18, 21, 23, 25–27) have shown that the upregulation of inflammation, oxidative stress, apoptosis, and DNA-damaged biomarkers were strongly associated with deterioration of heart function in setting of AMI (refer Figure 7). In this way, our findings were consistent with the findings from the previous studies (17, 18, 21, 23, 25–27). However, these parameters were markedly attenuated in the AMI-M group, once again explaining why hBMMSC treatment could reduce the infarct size/cardiomyocyte death and preserve LV function.

Angiogenesis has been identified to play a fundamental role in improving ischemia-related organ dysfunction (23, 25–27) through restoring the blood flow into the ischemic area. A principal finding in the present study was that the molecular levels of angiogenesis markers (refer Figure 6) were significantly increased in AMI-M than in AMI-B animals. Perhaps, these findings could also explain why the LVEF was greatly preserved, and the infarct size was markedly alleviated in the former group than in the latter group.

### Study limitation

Our study has limitations. First, this study did not provide the classification of heart failure because some specific parameters, including the symptom, sign, and physical examination which indicate heart failure, were not easily measured in porcine AMI. Second, although the results were attractive and promising, this study did not test the effect of different doses of hBMMSC therapy on improving the outcome after AMI induction. Thus, we have no comment for which dose of OmniMSCs is the optimal one for treatment of AMI. Third, although this study already provided serial changes of LVEF that were measured by transthoracic echocardiograph, a fly in the ointment in the present study is that we did not provide the cardiac MRI-measured parameters of LVEF, stroke volume, and systolic/diastolic dimension to more accurately evaluate the LV remodeling.

In conclusion, early intracoronary administration of the OmniMSCs was safe and effective in reducing infarct size and significantly preserving the LV function in porcine AMI.

## Data Availability

The raw data supporting the conclusions of this article will be made available by the authors, without undue reservation.
