# Supplementary figures and images for: Safety and efficacy of intracoronary artery administration of human bone marrow-derived mesenchymal stem cells in STEMI of Lee-Sung pigs—A preclinical study for supporting the feasibility of the OmniMSC-AMI phase I clinical trial
Source: Front Cardiovasc Med. 2023 Mar 29;10:1153428. doi: 10.3389/fcvm.2023.1153428 (PMC10091140; doi:10.3389/fcvm.2023.1153428)

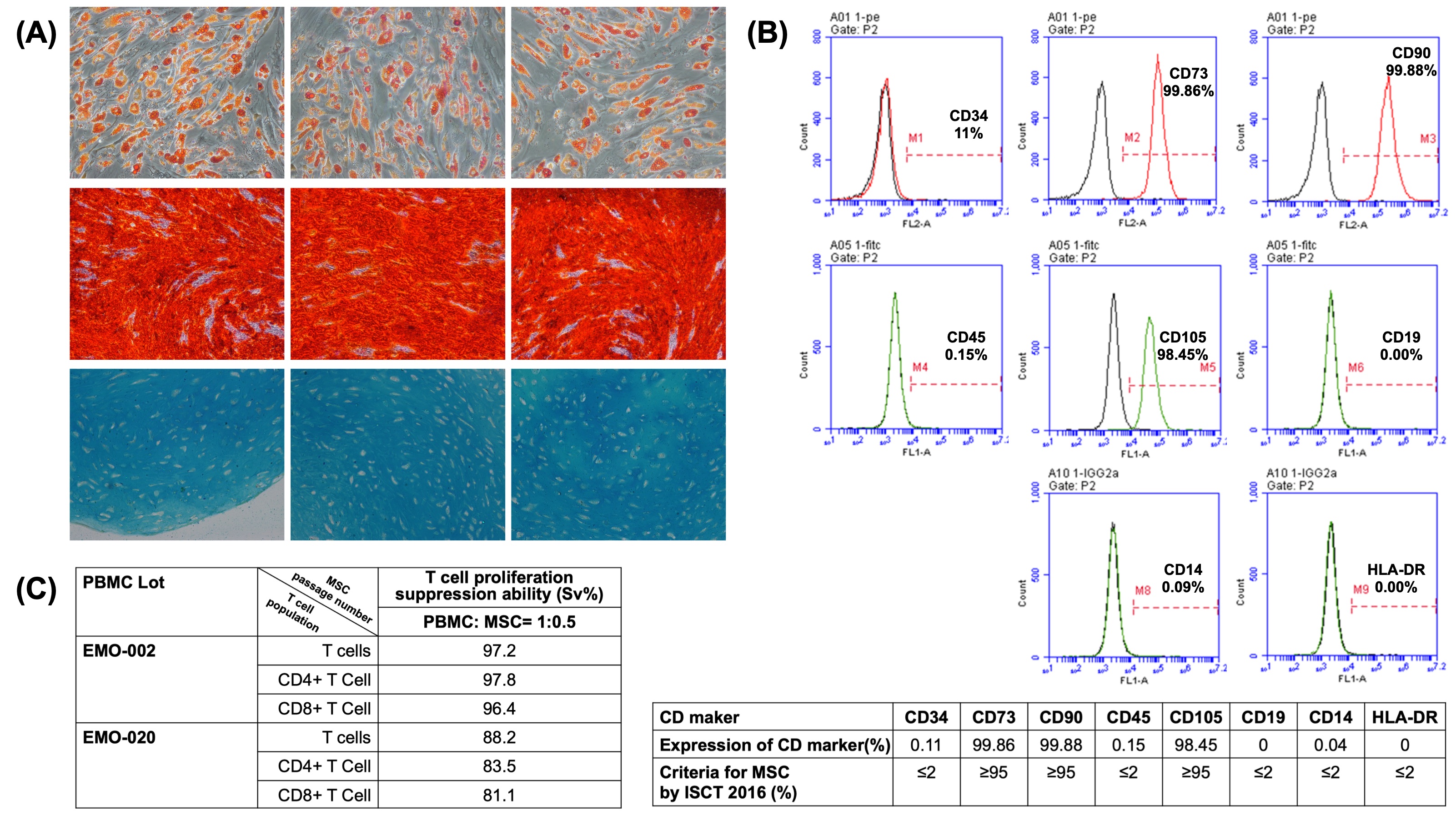

Supplement: Supplementary file 1 [file Image1.jpeg]

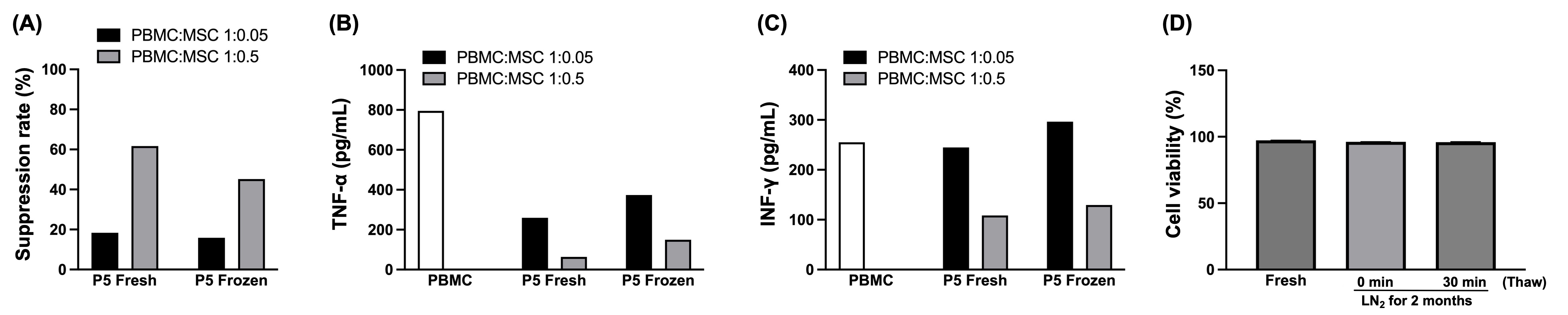

Supplement: Supplementary file 2 [file Image2.jpeg]
